# Sexual dimorphism in colorectal cancer: molecular mechanisms and treatment strategies

**DOI:** 10.1186/s13293-024-00623-1

**Published:** 2024-06-12

**Authors:** Yair Rodríguez-Santiago, Claudia Angelica Garay-Canales, Karen Elizabeth Nava-Castro, Jorge Morales-Montor

**Affiliations:** 1 Departamento de Inmunología, Instituto de Investigaciones Biomédicas, Universidad Nacional Autónoma de México, Coyoacán, Mexico City, 04510 México; 2 Posgrado en Ciencias Biológicas, Universidad Nacional Autónoma de México, Edificio D, 1er piso, Circuito de Posgrados, Ciudad Universitaria, Ciudad de México, 04510 México; 3 Grupo de Biología y Química Atmosféricas, Instituto de Ciencias de la Atmósfera y Cambio Climático, Universidad Nacional Autónoma de México, Ciudad Universitaria, CDMX, 04510 México

**Keywords:** Sexual dimorphism, Neuroimmunoendocrine network, Colon cancer, Sex steroids, Estrogen receptor, Androgen receptor

## Abstract

**Introduction:**

Sexual dimorphism significantly influences cancer incidence and prognosis. Notably, females exhibit a lower risk and favorable prognosis for non-reproductive cancers compared to males, a pattern observable beyond the scope of risk behaviors such as alcohol consumption and smoking. Colorectal cancer, ranking third in global prevalence and second in mortality, disproportionately affects men. Sex steroid hormones, particularly estrogens and androgens, play crucial roles in cancer progression, considering epidemiological in vivo and in vitro, in general estrogens imparting a protective effect in females and androgens correlating with an increasing risk of colorectal cancer development.

**Main body:**

The hormonal impact on immune response is mediated by receptor interactions, resulting in heightened inflammation, modulation of NF-kB, and fostering an environment conducive to cancer progression and metastasis. These molecules also influence the enteric nervous system, that is a pivotal in neuromodulator release and intestinal neuron stimulation, also contributes to cancer development, as evidenced by nerve infiltration into tumors. Microbiota diversity further intersects with immune, hormonal, and neural mechanisms, influencing colorectal cancer dynamics. A comprehensive understanding of hormonal influences on colorectal cancer progression, coupled with the complex interplay between immune responses, microbiota diversity and neurotransmitter imbalances, underpins the development of more targeted and effective therapies.

**Conclusions:**

Estrogens mitigate colorectal cancer risk by modulating anti-tumor immune responses, enhancing microbial diversity, and curbing the pro-tumor actions of the sympathetic and enteric nervous systems. Conversely, androgens escalate tumor growth by dampening anti-tumor immune activity, reducing microbial diversity, and facilitating the release of tumor-promoting factors by the nervous system. These findings hold significant potential for the strategic purposing of drugs to fine-tune the extensive impacts of sex hormones within the tumor microenvironment, promising advancements in colorectal cancer therapies.

## Highlights

Androgens, particularly dihydrotestosterone, are implicated in colorectal cancer risks due to their role in fostering an immunological milieu conducive to tumor growth diminishing anti-tumor cytotoxic responses, stimulating the release of catecholamines, serotonin, acetylcholine and growth factors from sympathetic and enteric nervous system and adversely impacting microbial diversity.

Estradiol confers colorectal cancer protection by attenuating inflammatory processes, enhancing microbial diversity, and suppressing tumor-promoting activities of the sympathetic nervous system through neurotransmitter regulation.

These insights pave the way for repurposing existing of new use of old antiandrogens like flutamide and employing estrogen receptor beta agonists in colorectal cancer treatment strategies, highlighting a potential shift in therapeutic approaches.

## Introduction

The regulation of physiological processes in the colon is closely related to these three systems. For example, the intestine is continuously exposed to antigens from food and the microbiota. Therefore, regulation of the immune system is crucial for maintaining homeostasis between immunosuppressors and the inflammatory process [1]. On the other hand, the bowel possesses the largest component of the autonomic nervous system, called the enteric nervous system, which is crucial for the physiological processes of the colon and the development of many gastrointestinal diseases. For instance, patients with ulcerative colitis and Crohn’s disease exhibit alterations in the number of enteric neurons and neurotransmitter synthesis [2].

Sex steroids also play a role in the physiology of the colon. Estrogens influence epithelial membrane permeability, serotonin production, tight junction expression, inflammation, and microbiome composition [3]. The role of androgens is poorly understood; however, in a mouse model, androgens alter the proliferation of enterocytes and increase the size of crypts [4]. Therefore, the interaction of sex steroids with the immune system, nervous system, and microbiota likely plays an essential role in explaining the sexual dimorphism observed in colorectal cancer (CRC) patients. The aim of this review was to analyze the effects of the principal sex steroids (estradiol, testosterone, and dihydrotestosterone) on CRC development and the regulatory effects they exert on the immune system, autonomic nervous system, and microbiota and to propose a model that represents the involvement of these molecules in tumor growth.

## Main text

### Sex steroids and colon cancer development: what do we know?

#### Epidemiology

Epidemiological data indicate that colorectal cancer (CRC) is the third most common cancer worldwide and the second leading cause of mortality. Risk factors for CRC include dietary habits, inflammatory bowel diseases, metabolic disorders, alcohol consumption, and tobacco smoking [5]. Interestingly, men have a greater incidence of CRC than women do, suggesting that sex is a contributing factor. This greater incidence can be attributed to various causes, categorized into sex and sexual differences, including disparities in behavior and physiological conditions. Moreover, sexual dimorphism is consistently observed across different ethnicities and geographical locations, indicating that intrinsic factors play a significant role in explaining this phenomenon. One of the key intrinsic factors that differs between the sexes is the concentration of sex steroids, although not the only factor. Supporting this idea, women between 18 and 44 years old have a higher survival rate than men of the same age or older women (over 50 years old) [6]. Interestingly, women have a greater risk of develop tumors in proximal colon than men. Also, the incidence of CRC in proximal colon increases with age; in men, the effect of age is less significant [7]. These data suggest that estrogens protect against CRC in younger women against the develop of left-sided colon neoplasia. In line, the exposition to estrogens by hormonal replacement therapy or pregnant reduce the risk of microsatellite instability, which is more frequently in left-site compared with right-sided colon tumors [8].

Compared with healthy individuals, CRC patients exhibit dysregulation of serum sex steroid levels, although the exact role of sex steroids has not yet been determined. For instance, postmenopausal women with CRC have higher levels of estradiol (E2) and estrone (E1) and a greater ratio of testosterone (T) to E2 than control patients do; however, this pattern is not observed in men [9, 10]. In normal and neoplastic colon tissue, the expression of estrogen receptor beta (ER-β) is predominating respect with ER-α and their expression is loss in advance stages of CRC disease. Also, the loss of expression is associated with poorer survival rate in both sexes [11]. These data suggest that estrogens protect against CRC due their interaction with ER- β. In line, postmenopausal women with hormonal replacement therapy have a lower risk of develop CRC in ER-β positive cases but not in ER-β negative [12]. Also, in preclinical studies with animals deficient of ER- β increase the number and size of colon tumors [13]. On another hand, the higher expression of ER-α is associated with poor prognosis (less overall survival, tumor differentiation, tumor invasion, lymph node status and Dukes stag) [14, 15]. When ER- β is expressed, E2 reduce the ER- α protein levels which could explain the predominance of ER- β in normal colon epithelium and that in neoplastic tissue there is a reduction of ER- α [16].

Moreover, men exhibit an increase in the level of ER-α [17]. Conversely, the expression of aromatase, an enzyme responsible for converting T to E2, is greater in neoplastic tissue in both men and women, and this expression is associated with the proliferation index, estrogen concentration, and lower survival rate [10]. Consistently, intratumoral levels of E2 are greater in male patients with CRC than in healthy controls [18].

The relationship between androgens and CRC has been poorly studied. Contrary to expectations, the circulating level of T is inversely associated with overall survival and mortality in CRC patients but only in men [19, 20]. A reduction in androgen levels is associated with fewer CAG repeats in the androgen receptor (AR) gene, which subsequently increases transcriptional activity [21]. AR is overexpressed in colon tumor tissue and is associated with tumor size, differentiation, and distant metastasis [22]. In contrast, this receptor is not expressed in the nonneoplastic mucosa. Interestingly, in postmenopausal women, a few CAG repeats in the AR gene and a CA repeat in the ER-β gene are associated with high serum androgen levels, suggesting that these polymorphisms have a stimulatory effect on T production in women [23]. Although further research is needed, deregulation of sex steroid concentrations, expression of sex steroid receptors, and enzymes involved in sex steroid metabolism have been observed in CRC patients (Table 1).

**Table 1:** Epidemiological, in vivo and in vitro studies about the role of sex steroids in colorectal cancer development. Arrow up indicates stimulation and arrow down inhibition

SEX STEROIDS	EFFECT
**Estrogens.** **Epidemiological studies** [9–11, 18, 24]	⇩Incidence of CRC in women than men. ⇧E2, E1 and T/E2 concentration in postmenopausal women, but not in men. ⇩ERβ in tumor⇧cancer stages and tumor invasion. ⇧Aromatase in tumor ⇧Estrogens level ⇧Intratumoral E2 levels in men than healthy tissue.
**In vivo studies** [25–29]	⇧E2 ⇩Colon tumors in male and females. ⇧E2 ⇧⇩ Genes related with NF-kB, NRF2 and NLRP3. ⇩ERβ ⇧Inflammation and tumor growth.
**In vitro studies** [30–36]	⇧E2 ⇩Viability and migration ⇧E2 ⇧Apoptosis ⇧E2 ⇧⇩ Modulate P38/MAPK, JNK/PGE2, PI3K/AKT, MYC, MYB, RUNX2 and PROX1 pathways ⇧ ERβ Modulate the action of E2.
**Androgens.** **Epidemiological studies** [19–23]	⇧T in men ⇩ survival ⇩T in men ⇩CAG repeats in AR gene ⇩CAG in AR and CA in ER ⇧T levels ⇧AR in tumor tissue ⇧Tumor stage and metastasis
**In vivo studies** [26, 37–39]	⇧Androgens ⇧Number and size of tumors ⇧Androgens ⇧Inflammation (iNOS, COX2 and NRF2) ⇧Androgens ⇧Apoptosis in tumor cells
**In vitro studies** [39, 40]	⇧T ⇧Apoptosis and migration ⇧T⇧cytoskeleton reorganization PI3K/AKT/JNK pathway.

Therefore, these genes may be potential markers for CRC, which is an important step in understanding the dimorphic nature of this neoplasia. This finding allows for the proposal of more targeted treatments based on sex. These associations suggest that sex steroids play a role in the development of CRC and that their effects differ between men and women. Thus, to elucidate the mechanisms through which sex steroids influence the development of CRC, it is necessary to analyze both in vivo and in vitro studies.

#### In vivo

According to epidemiological data, different animal models, such as ICR (outbred mouse), C57BL6 (endogamous mouse), and PIRC (rats naturally susceptible to developing adenomas), develop more and larger tumors than their female counterparts. Various analyses have been conducted to elucidate whether sex steroids play a role in this susceptibility and to determine their specific effects. The azoxymethane (**AOM**)/dextran sodium sulfate (**DSS**) model of colitis-associated cancer is a consistent, reproducible, and relatively inexpensive initiation-promotion model that utilizes chemical induction of DNA damage followed by repeated cycles of colitis [41]. Compared with control female, ovarietomized (OVX) female ICR mice treated with AOM/DSS developed more tumors but of equal size, and reconstitution with E2 inhibited this effect [42]. It is important to note that tumor size is important in colorectal cancer (CRC) because it is associated with cancer stage, tumor aggressiveness, and distant metastasis [43]. Despite this, OVX animals exhibited greater tissue damage than control animals, which was not observed in the reconstituted group, and only animals treated with E2 presented lower concentrations of IL-6, Cox2, and TNF-α [42]. In contrast, in PIRC rats, neither ovariectomy nor reconstitution with E2 influenced the number of adenomas [26]. These findings suggest that E2 participates in the maintenance of colon architecture but has a minor role in the pathophysiology of CRC in females.

Interestingly, E2 appears to have different effects on males and females. Male ICR mice had more tumors than male mice treated with E2 and females. Additionally, treatment with this hormone significantly reduce the transcription of genes involved in tumor progression pathways, such as NF-κB, NRF2, and NLRP3 ^25^. NRF2 is a crucial in the response to oxidative stress under normal conditions and participate in the colorectal cancer progression. In AOM/DSS model NRF2 knockout mouse increase the incidence in tumor formation that is associated with an elevated oxidant factors such as COX-2 [44]. In vitro studies shown that the activation of NRF2 inhibits the activation of NF-κB increasing apoptosis in colon cancer cell lines [45]. Interestingly, the expression of the genes of these pathways (NF-κB, NRF2, and NLRP3) is different between males and females only when treatment with AOM and DSS is administered and not basally. In early stages of induction of tumors (2 weeks) males have higher levels of NF-κB related genes (iNOS, COX-2, IL-6 and TNF-α) than females and lower levels of NRF2 and some related genes of antioxidant factors (NQ-O1). This suggest that early inflammation help to the susceptibility in males. When E2 is administrated in males, the levels of NF-κB related genes diminish and the expression of NRF2 related genes increase it [25]. These results indicate that E2 reduces inflammation from the early stages of colorectal tumor development. On another hand, in advance stages of the induction of tumors (10 weeks) males have higher levels of NRF2 and its related genes than males treated with E2 and females. These suggest a double role of these pathways in the pathogenesis of CRC [25]. This is in line with other studies that suggest that the activation of NRF2 specifically in tumors cells increase their tumorigenicity, survival, growth and chemoresistance [46]. In the case of NLRP3, males exhibit greater expression of NLPR3-related genes (IL-1β and IL-18) than males treated with E2 and females. IL-1β and IL-18 trigger a chronic inflammatory process promoting the formation of tumors [47]. The treatment with E2 in males results in a downregulate expression of NLPR3 and its effectors (IL-1β and IL-18) [25].

Treatment with T has different effects on androgen levels. Orchiectomy (ORX) in C57BL6 mice drastically reduces the number and size of induced tumors. Intraperitoneal propionate of T slightly increased the number of tumors in males and the size only in females, but it did not completely reverse the effect caused by orchiectomy. At the molecular level, factors associated with the NFκB and NRF2 pathways, such as iNOS, COX-2, and NRF2, are expressed at lower levels in ORX mice and females than in control males [37].

On the other hand, in the AOM/DSS-treated BALB/c mouse group and in the APC+/- mouse group (unspecifed sex), mice subcutaneously injected with a complex of T-albumin (the target membrane androgen receptor) developed significantly fewer tumors. These animals had lower levels of p-Akt and p-Bad than did the control animals. Additionally, treated mice exhibit more apoptotic cells than healthy controls [38, 39]. The choice of study model and the analysis by sex are important factors to consider when elucidating the role of T; moreover, its metabolism could be considered since T can be converted into E2 or DHT. Interestingly, in ORX PIRC rats reconstituted with DHT, the protective effect of gonadectomy is inhibited [26]. These findings suggest that castration of animals protects against tumor induction due to the bioavailability of DHT.

Consistent with the idea that T could have a protective effect, a lower concentration of T in CRC patients is associated with lower survival and mortality. There are two possible explanations for this difference. First, T is converted to E2, which has a protective effect. The second explanation is that its interaction with the membrane androgen receptor triggers an antitumoural role, which is supported by the in vivo studies analyzed in this section. There is clear modulation of tumor growth by sex steroids (Table 1).

However, additional data are needed to draw definitive conclusions. While estrogen appears to participate in the maintenance of colon architecture and plays a minor role in CRC pathophysiology in females, it has a more drastic protective effect in males. On the other hand, the role of androgens in CRC is complex and dependent on the model and analysis by sex. Orchiectomy provides protection against tumor induction, and T seems to exert a protective effect mediated by the membrane androgen receptor or its conversion to E2.

#### In vitro

E2 reduces the viability of many colon cancer cell lines and alters important processes, such as migration, motility, and apoptosis. For example, this hormone reduces the viability of colon cancer cells by activating P53, which subsequently upregulate the levels of p21 and p27, that consequently inhibits cyclin D1 gene that reduce proliferation [30]. The reduction in viability of DLD-1 cells caused by incubation with E2 was inhibited by palmitoylation inhibitors such as 2-Br-palmitate. Palmitoylation is the process of biding a protein to and fatty acid and this led the attachment of proteins (such as estrogen receptors) to the plasma membrane. 2-Br-palmitate inhibit fatty acid CoA ligase and other enzymes that reduce the levels of intracellular palmitoyl-CoA [48]. ER-β activates p38 downstream pathway when is binding to the membrane, however If palmitoylation is inhibited, this process does not occur. This finding suggested that the effect of E2 is mediated through a nongenomic pathway involving the activation of the P38/MAPK pathway [31]. Additionally, E2 treatment decreases cellular migration by reducing MMP-2 and MMP-9 levels and inhibiting the JNK 1/2/PGE2 pathway, which is involved in cellular motility [32]. In recent years, researches with a metabolite of E2, 2-methoxyestradiol, indicates thar regulate several biology processes such as proliferation and shown anticancer properties [49]. In this case, this metabolite is capable of increasing apoptosis colon cancer cells by upregulating proapoptotic factors such as P53, Bax, p21, and caspases 3 and 9 and decreasing antiapoptotic factors such as c-Myb and bcl-2 ^33–35^. Moreover, blocking the p38 signal reduces the expression of ER-β, indicating that rapid (nongenomic) is necessary for this slow effect (genomic effect). Experiments with microarrays have shown that ER-β plays an important role in the regulation of transcription factors such as MYC, MYB, RUNX2, and PROX1, which are involved in cell viability, proliferation, apoptosis, and differentiation. This modulation potentially triggers an antitumoral cascade [36].

Androgens, particularly T, also regulate processes in colon cancer cells. Treatment with this hormone stimulates the reorganization of actin filaments, and interestingly, flutamide does not inhibit this effect. Flutamide inhibits the bind of androgens just with cytoplasmatic androgen receptors, suggesting that the pathway is mediated through the membrane androgen receptor [50]. First, T increases FAK protein levels, which triggers a signaling cascade that stimulates actin reorganization. FAK overexpression regulates survival, cellular adhesion, motility, and proliferation, and in cancer, it is associated with advanced stages and metastasis [51]. On the other hand, cytoskeletal reorganization is a marker of early apoptosis in a very complex process [52]. Just an example, the reorganization actin-membrane linker protein ezrin activate CD95 that activate apoptosis, disruption of microfilaments by cytochalasin D induce the translocation of proapoptotic factors, among other [53]. Similarly, T increases apoptosis in CaCo2 cells by reducing PI3K/Rac1, which activates the kinase JKN, stimulating the transcription of proapoptotic genes involved in intrinsic and extrinsic apoptosis [40, 54]. Finally, reorganization of the cytoskeleton reduces the migration and invasion of colon cancer cells. This effect is not influenced by the inhibition of aromatase, suggesting that the effect is directly mediated by T and not by the conversion of T to E2 ^50^; since, this enzyme metabolizes T to E2 and E2 is a final metabolite in the steroidogenesis.

As mentioned above, there are close interactions among the three macrosystems present in the colon. In this section, we analyze certain aspects of the regulatory effect of sex steroids on the development of colorectal cancer. However, most related studies have focused only on analyzing the effects of certain factors on the immune system and have not conducted deeper examinations. Furthermore, these studies failed to explore the effects on the enteric nervous system, which is crucial for obtaining a complete understanding of this neuroimmunoendocrine interaction. Consequently, in the subsequent sections, we will examine the impact of estrogens and androgens on cells of the immune system, the enteric nervous system, and the intestinal microbiota, all of which maintain a close relationship with these three macrosystems.

#### The role of inflammation in the development of colorectal cancer: a general overview

Inflammatory bowel disease (IBD) has an increased risk of develop CRC. The cumulative risk is dependent of the years with the disease 1%, 3%, and 7% at 10, 20, and 30 years, respectively, while, sporadic is the most common type of CRC [55]. However, some mutation and especially the need for a chronic inflammatory process are similar in both types. In this review, we focus on the research focus in process that occur in IBD patients and in the AOM and DSS animal model, which is more like CRC associated with colitis than to sporadic CRC [56].

**Fig. 1:**
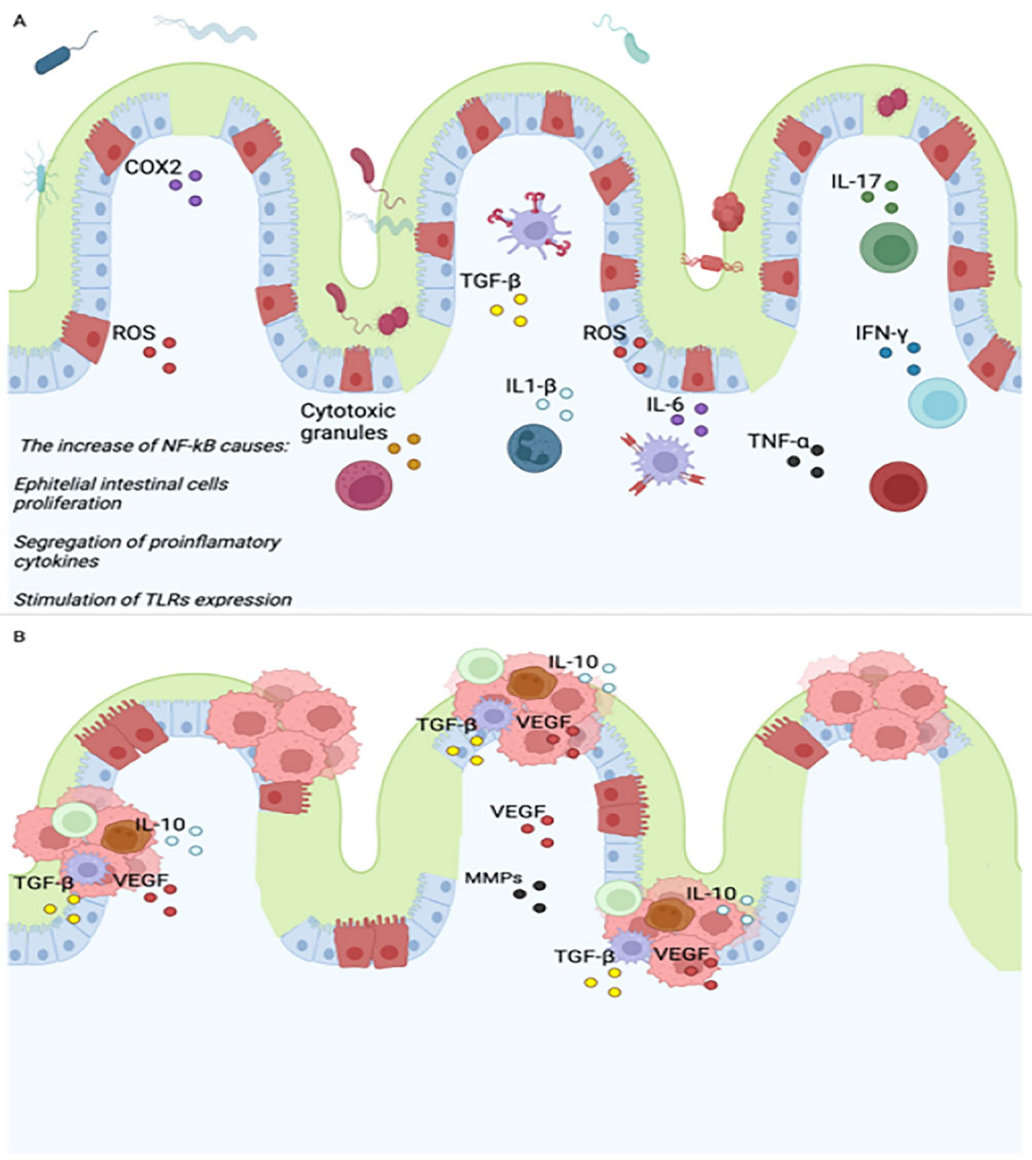
Pathogenesis of colorectal cancer associated with ulcerative colitis (early stages of CRC). Innate cells and adaptative immune cells increase their production of soluble factors which can trigger a chronic inflammation process. If this state is maintained, the possibility of developing cancer is greater due to an increase in the mutation rate and the loss of intestinal homeostasis. One of the pathway involved in this process is NF-kB that contributes increasing the proliferation of intestinal cells, modulating the production of proinflammatory cytokines and stimulating the expression of receptors such as TLRs and MCH II (**A**). On another hand, in advance stages of cancer, immune cells change to an immunosuppressor phenotype. Both tumor and immune cells secrete factors that hinder the effective elimination of transformed cells (**B**). abbreviations: ROS, reactive species of oxygen. COX2, cyclooxygenase 2. NF-kB, nuclear factor kappa-light-chain-enhancer of activated B cells. IL-(6,10, 1β), Interleukin. TGF-β, Transforming growth factor beta. TNF-α, tumor necrosis factor alpha. MHC, Major Histocompatibility Complex. TLR, Toll-like receptors. VEGF, Vascular endothelial growth factor. MMPs, metalloproteinases. This figure was created with BioRender.com

Figure 1A represent the early stages of CRC that is characteristic by chronic inflammation where cells of the innate and adaptive immune response participate and promotes the development of tumors growth.

The first cells involved early in pathogenesis are myeloid cells, primarily neutrophils and macrophages, as well as natural killers that increase the production of reactive oxygen and nitrogen species (ROS and NOS), cytotoxic granules, and proinflammatory cytokines [57]. Chronic states of inflammation, such as those observed in patients with IBD, contribute to tissue damage, loss of the epithelial membrane, ulcer formation, and an increased mutation rate, potentially leading to tumor formation. IBD patients exhibit high infiltration of myeloid cells that perpetuate the chronic inflammatory process. The activation of NF-κB is one of the principal mechanisms influencing the proinflammatory environment, as it regulates the release of proinflammatory molecules such as TNF-α, IL-1β, IL-6, iNOS, and ROS, which are elevated in the serum of patients with ulcerative colitis. TNF-α promotes the survival of epithelial cells, the release of protumoral cytokines, and direct disruption of the epithelial barrier (Fig. 1A).

Moreover, the inhibition of TNF receptors reduces tumor formation and the infiltration of neutrophils and macrophages in mice with tumors induced by azoxymethane (AOM) and dextran sulfate sodium (DSS). IL-6 and IL-1β promote proliferation and survival by activating the transcription factor STAT-3 while downregulating the key protein P53, leading to an increased mutation rate. Animal models have shown that IL-6 enhances tumor growth associated with colitis, and this effect can be inhibited by blocking IL-6. ROS and iNOS cause tissue damage and direct DNA damage and stimulate the secretion of proinflammatory cytokines, thereby promoting inflammation and increasing the mutation rate [58] (Fig. 1A).

Adaptive immune cells also participate in the progression of ulcerative colitis toward colon tumors. Dendritic cells connect the innate immune response with the adaptive immune response. In the basal state of the epithelium, these cells exhibit an immunosuppressive phenotype and primarily secrete anti-inflammatory cytokines such as IL-10. Inflammatory bowel disease (IBD) patients exhibit increased infiltration of DCs and increased expression of TLRs, which help activate NF-κB and subsequently promote an inflammatory microenvironment [58]. Conversely, DCs stimulate the migration and differentiation of lymphocytes. IBD patients exhibit increased infiltration of activated lymphocytes. CD8 + lymphocytes directly kill tumor cells by secreting cytotoxic granules and proinflammatory cytokines (TNF-α and IFN-γ). The inhibition of costimulatory molecules that activate these cells (CD80) in AOM/DSS models increases tumor formation [57] (Fig. 1A).

On the other hand, CD4 + lymphocytes play a determinant role in regulating the growth or elimination of transformed cells; however, their function depends on their phenotype. TH1 cells assist the innate immune response in killing neoplastic cells. The Th2 response inhibits the Th1 response by releasing IL-13, IL-21, and IL-25. IL-13 affects epithelial membrane integrity, inducing apoptosis in epithelial cells and preventing their regeneration. The increase in TH2 cells depends on the IL-33 concentration, which is elevated in IBD patients. TH17 cell infiltration is increased in IBD patients and promotes a proinflammatory microenvironment by directly releasing and/or stimulating the production of cytokines such as TNF-α, IL-6, IL-17, IL-22, and IL-21. Blocking IL-21 reduces tumor formation and the production of proinflammatory cytokines (Fig. 1A).

Among Tregs, the phenotype CD4 + Foxp3 + cells inhibit tumor growth in AOM/DSS models, IL17 + Foxp3 + CD4 + T cells are present in higher concentrations in IBD patients, while Foxp3 + RORγt + T cells potentially aid in tumor progression through the secretion of IL-17, favoring an inflammatory microenvironment, and have a greater capacity to inhibit the cytotoxic response of CD8 + lymphocytes, thereby preventing the elimination of transformed cells [57–59] (Fig. 1A).

The microenvironment in colon carcinoma differs from that in dysplasia in IBD patients. Both tumor and immune cells secrete factors that hinder the effective elimination of transformed cells. These cells primarily maintain an immunosuppressed state through the synthesis of anti-inflammatory cytokines (TGF-β and IL-10), express inhibitory receptors such as CTL-4 and PD-1 and secrete factors that modulate angiogenesis and disrupt the extracellular matrix, such as VEGF and MMPs. These factors enable cells to invade other tissues and eventually reach other organs, primarily the liver and lungs [60] Fig. 1B.

#### General mechanisms of sex steroids in the immune system

Estradiol (E2), testosterone (T), and dihydrotestosterone (DHT) are steroid hormones that have both genomic and nongenomic effects on immune cells. These hormones can bind to specific nuclear receptors to regulate gene transcription and protein synthesis. On the other hand, the nongenomic effects of these hormones occur through their interaction with cell membrane receptors, triggering rapid signaling cascades.

**Fig. 2:**
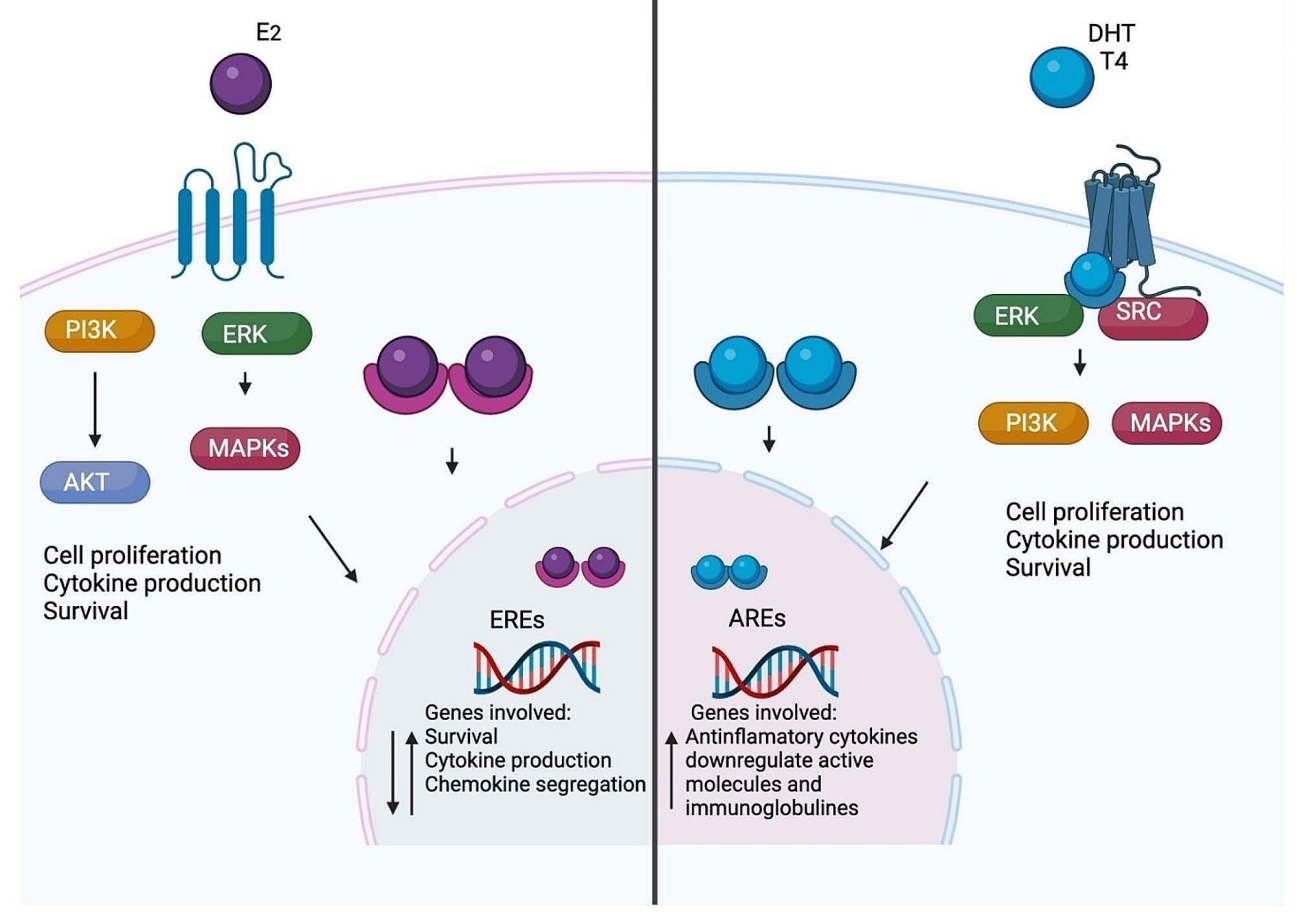
General action mechanism of sex steroids. These molecules can exert their effects on cells through binding to cytoplasmic receptors (genomic effect). These receptors translocate to the nucleus and binds to EREs or AREs. The other mechanism is through membrane receptors (non-genomic effect) that activates signaling cascades such as PI3K/AKT, MAPKs and ERK. Both mechanisms modulate process involved on inflammation such as survival, cytokine production, chemokine segregation. The effect of estrogens depends principally on the dose and the type of receptor expressed (alpha or beta) (**A**). On another hand, in general, androgens exert and immunosuppressor phenotype (**B**). abbreviations: PI3K: phosphoinositide 3-kinases. MAPKs: mitogen-activated protein kinases. EREs: estrogen response elements. AREs: androgen response elements. ERK: extracellular signal-regulated kinase. AKT: alpha serine/threonine-protein kinase. SRC: proto-oncogene tyrosine-protein kinase. E2: estradiol. T: testosterone. This figure was created with BioRender.com

In the Fig. 2, we represent the genomic and non-genomic effect of E2, T and DHT and their general influence on immune response. In general, E2 can have a dual role depending of the dose, the tissue and the specific cell target. While, androgens have, in general, an immunosuppressor growth that could be favoring the growth of tumor (Fig. 2).

E2 exerts its genomic effects by binding to estrogen receptors (ER-α and ER-β), which are expressed by several types of immune cells, including T cells, B cells, dendritic cells, natural killer cells, monocytes, neutrophils, and macrophages. When estradiol binds to ERs, it dimerizes and translocate to the nucleus, where it binds to estrogen response elements on target gene promoters and regulates transcription. For example, estradiol upregulates the expression of genes involved in survival (*cd22, shp-1, bcl-2*, and *vcam-1*), cytokine production (TNF-α, IL-6, IL-1β, and IL-10), and chemokine segregation (CINC-1, CINC-2β, and CINC-3), among others. Additionally, E2 can regulate the expression of transcription factors that modulate the expression of immune-related genes, such as NF-κB [61, 62] (Fig. 2).

T and DHT exert their genomic effects by binding to androgen receptors (AR), which are expressed by several types of immune cells, including T cells, B cells, dendritic cells, monocytes, neutrophils, and macrophages. Upon binding to testosterone or DHT, as described above, ARs dimerize and translocate to the nucleus, where they bind to androgen response elements on target gene promoters and regulate transcription. For example, T upregulates the expression of genes involved in anti-inflammatory cytokine production (IL-10 and TGF-β) and downregulates the expression of activated molecules (MHC-1 and CD86) and immunoglobulins [63] (Fig. 2).

In addition to their genomic effects, E2, T, and DHT also exert nongenomic effects on immune cells through their interaction with cell membrane receptors, such as G protein-coupled receptors. E2 can activate the MAPK and PI3K/Akt signaling pathways through its interaction with GPCRs, which in turn can regulate T-cell proliferation, survival, and cytokine production [64]. Similarly, cytoplasmic androgen receptors can activate intracellular signaling pathways such as the ERK/MAPK and AKT pathways. Furthermore, androgens interact with and affect the membrane receptor GPRC, activating a signaling pathway that enhances or diminishes survival, proliferation, cytotoxic effects, and the segregation of proinflammatory (IL-1β, IL-6, and TNF-α) or anti-inflammatory (IL-10 and IL-4) cytokines [63, 65] (Fig. 2).

Consistently with the modulation of sex steroids on immune system, aging and sex are an important factor that influence immune system and repercuss in inflammation. Young females have more activated immune cells than males. For example, dendritic cells that produces IFN-γ, activated macrophages, greater cytotoxicity T cell activity and highest antibody production [66]. In postmenopausal women increase the production of serum proinflammatory cytokines such as IL-6 and TNF-α, which are cytokines that can promote chronic inflammation and exacerbate carcinogenic process, and also present a diminish in activated NKs [67]. Concordantly, as we discussed, epidemiological studies suggest that estrogens exert a protection to develop right-sides colon tumors in young women. One possibility is that, estrogens increase the infiltration or activation of immune cytotoxic cells that kill tumor cells, since these cells are more abundant in the right side of colon [68]. However, there are some questions that have not been answered: Are the components in the tumor microenvironment between the left and right colon sides inherently different or depends of sex steroids concentration? Are the components of both sides of the intestine inherently different between males and females? ¿How sex steroids modulate the infiltration and activation of immune cells in colon tumors?

In the case of androgens, in general increase the immunosuppressor role that could contribute to the development of tumors in men. For example, reduce the activity of dendritic cells and increase the levels of T regulator and Myeloid-derived suppressor cells that avoid the annihilation of malignant cells [69]. In line, the expression of proinflammatory cytokines (TNF-α, IL-17 and IL-1β) and the abundance of activated T cells is lower in gut from males than females [70]. However, there is no evidence of how aging and androgens regulate the infiltration of immune cells in sex difference of pathophysiology of CRC (Fig. 2).

Although the mechanisms by which sex steroids affect the immune system are well studied, sex steroids have an effect and a close bidirectional relationship with cells of the nervous system and the intestinal microbiota. The effects and implications of these three macrosystems for the development of colorectal cancer are extensively analyzed in the following sections.

**Nervous system and colorectal cancer**.

The enteric nervous system (ENS) has been referred to as the “second brain” due to its ability to control independent functions of the intestine. It consists of plexuses and nerve fibers distributed throughout the various layers of the intestine. The nervous system directly influences the progression of cancer. The infiltration of nerves into tumors is known as perineural invasion. This innervation occurs in approximately 20% of patients and is associated with tumor stage, survival, invasion of adjacent tissues, metastasis, and poor prognosis [71]. Cancer cells migrate through nerves upon the release of growth factors that activate prosurvival signals in cancer cells and other cells within the microenvironment [72].

**Fig. 3:**
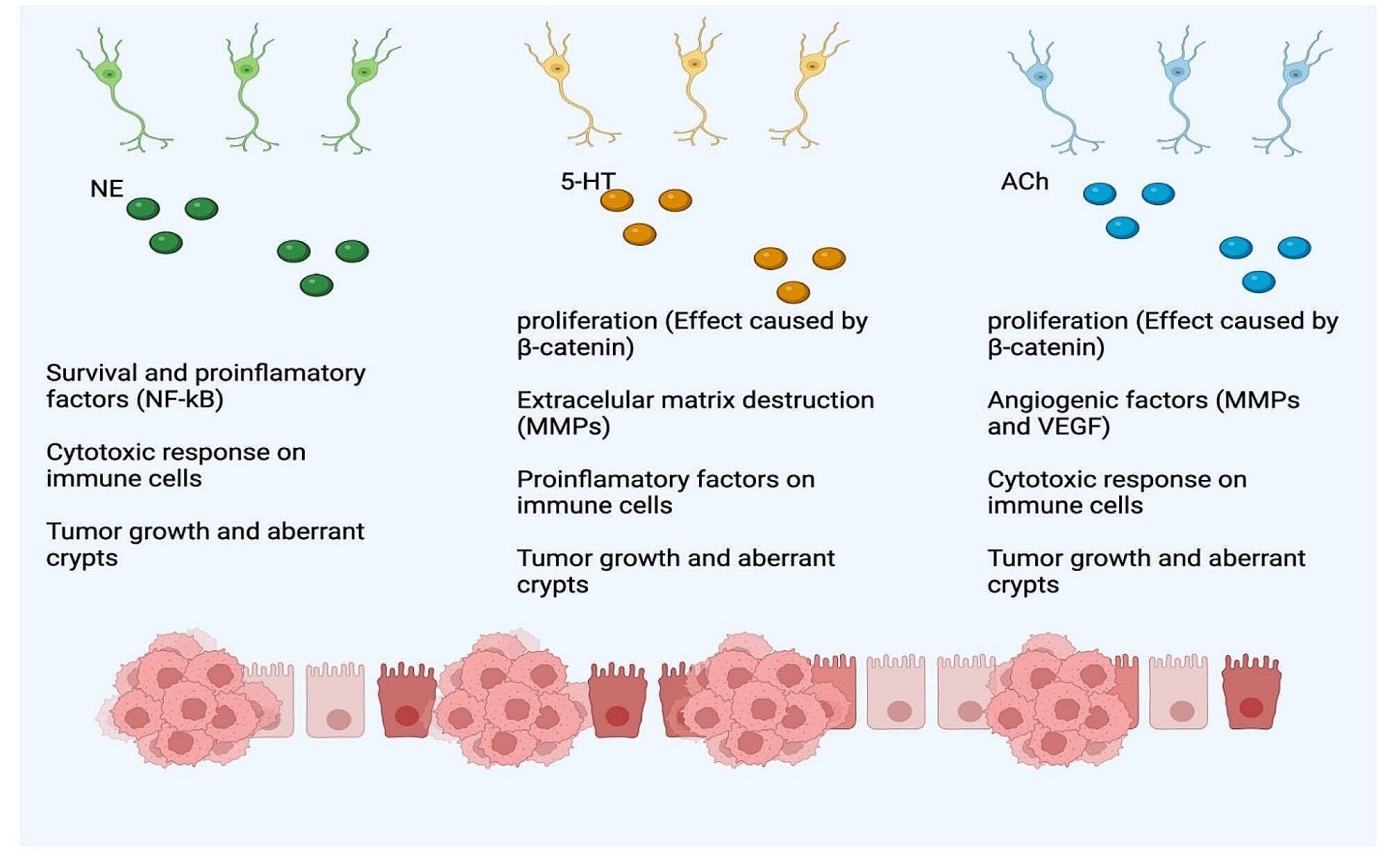
Mechanism of action of nervous innervation in the colon and tumor progression. CRC patients exhibit increased infiltration of nerve fibers directly associated with the stage of disease. Nerve fibers secrete neurotransmitter that influence increase inflammation, proliferation and invasion that promotes the development of tumors. These effects are carried out by the segregation of neurotransmitters such as NE, 5-HT and Ach that interact with their receptors in immune and tumor cells. NE: norepinephrine. 5-HT: serotonin. Ach: acetylcholine. MMPs: metalloproteinases. VEGF: vascular endothelial growth factor. This figure was created with BioRender.com

As represented in Fig. 3, the role of ENS exerts its effects through two mechanisms: the release of certain neuromodulators, primarily acetylcholine (Ach) and serotonin (5-HT), or direct stimulation of intestinal primary neurons. These factors regulate different processes, such as motility, permeability, secretion, blood flow, and proliferation of intestinal cells; the alteration of these process favor the development of CRC [73] (Fig. 3).

5-HT is produced by various cells in the intestine, including enterochromaffin cells in the villi, enteric serotonergic neurons in the myenteric plexus, mast cells, and even tumor colon cells [74, 75]. The concentration of this hormone is increased in patients with colorectal cancer (CRC) and is directly associated with cancer stage and decreased survival [76] (Fig. 3).

The concentration of tryptophan, which can be converted into serotonin (5-HT), is significantly reduced in patients with this neoplasia [77]. In mice with chemically induced colon tumors, inhibition of the enzymes involved in this conversion by pCPA reduces the number and size of neoplastic lesions. The same effect occurs when intestinal stem cells are modified to prevent the expression of SERT, THP1, THP2, and HTR1B in C57BL6 mice [76, 78]. 5-HT stimulates the expression of β-catenin through the APC pathway in tumor colon cells, initiating carcinogenesis [76]. It also increases the production of IL-1β in macrophages, stimulates the activation of the NLPR3 signaling pathway involved in inflammasome formation, and enhances the concentration of angiogenic factors (MMP-3 and 9) in AOM/DSS-induced colitis C57BL/6J mice (Fig. 3).

Interestingly, some studies have shown that the interaction between these two proteins is complex. For example, mice with intestinal stem cell deficiency in HTR2B cells have larger tumors due to the inhibition of the TGF-β/SMAD pathway and the activation of the IL-6 STAT3 pathway [79]. Additionally, an antidepressant drug (sertraline) that affects the 5-HT pathway inhibits tumor growth in xenografted mice, and fluoxetine decreases dysplastic aberrant intestinal crypts in rats [80, 81]. 5-HT may play a dual role in the pathogenesis of CRC.

Acetylcholine (Ach) is produced by enteric neurons and intestinal nonneuronal cells, such as CD8 + cells, CD4 + cells, NK cells, epithelial cells, and tumor colon cells [82, 83]. The Ach receptor is overexpressed in the mucosa of CRC patients and is directly associated with the disease stage and innervation of sympathetic nerve fibers and indirectly associated with the prognosis of CRC [84]. Compared with wild-type animals, mice deficient in the Ach receptor exhibit a decreased number and size of tumors, and their epithelial cells exhibit a decreased proliferation and death rate [85]. Similarly, in an orthotropic mouse model treated with a muscarinic receptor blocker, the number and size of tumors were drastically decreased (Fig. 3).

This chemical reduces the death marker PDL-1 and angiogenic factors and increases the expression of the immunoregulatory receptor FOXP3+, which is associated with a poor prognosis in many types of cancer due to the infiltration of T regulatory cells that prevent tumor destruction and increase the concentration of immune-related antitumoral cells, such as CD8+, CD4+, and neutrophils. Additionally, it reduces the expression of muscarinic receptors and the angiogenic factor VEGF [86]. As expected, Ach increases colon cancer cell proliferation through MAPK/ERK and PI3K signaling [87]. Its interaction with its receptor (AchR) also increases the release of MMP-9, which favors destruction of the architecture of epithelial tissue and invasion of colon cancer cells through the degradation of collagen IV [85, 87] (Fig. 3).

5-HT has a complex role in modulating immune cells that express 5-HT receptors. First, this hormone induces the secretion of proinflammatory cytokines (IL-1β, IL-6, and IFN-_γ_), inhibits the production of other cytokines, such as TNF-α, and suppress the NK cell capacity in monocytes, macrophages, and dendritic cells. They increase the recruitment of neutrophils but reduce their phagocytic and oxidative responses. Regarding the adaptive immune response, 5-HT increases cell proliferation and the production of IFN, thereby enhancing the immune response [88]. Specifically, in a DSS-induced colon model, 5-HT exacerbates the induction of colitis through an inflammatory response mediated by proinflammatory cytokines. It increases the release of IL-12 from DCs, which activates T cells that produce IL-17 and IFN-γ. In addition, macrophages in the tumor microenvironment express high levels of 5-HT receptors, and a selective antagonist of this receptor inhibits the synthesis of IL-12 and iNOS [89] (Fig. 3).

Acetylcholine (Ach) also regulates immune response. Activation of the Ach pathway in macrophages and neutrophils increases proliferation and the release of inflammatory mediators. This neurotransmitter also enhances the production of cytokines and chemokines in DCs, which stimulate the activation of T cells. Moreover, agonists of Ach receptors increase the proliferation rate of T lymphocytes through IL-2 and Ca^2+^ release. Animal models with deficiencies have shown that the elimination of tumor cells and the immune response against infections are increased by the activation of muscarinic receptors. In the case of B lymphocytes, activation of the muscarine receptor increases the production of IL-6 and immunoglobulins [7. Taken together, these findings suggest that Ach exerts its protumoral effect in mice through activation of the inflammatory response (Fig. 3).

In addition to autonomic nerve fibers, both sympathetic (affected by adrenaline and noradrenaline) and parasympathetic nerves (affected by acetylcholine) innervate colon tissue. In particular, the sympathetic nervous system is associated with the development of CCR. β-blockers, which inhibit adrenaline and noradrenaline receptors, have been shown to reduce the mortality rate and improve progression-free survival [90]. In vivo, denervation of both parasympathetic and sympathetic nerves decreases the number of aberrant crypts (early stages of CRC). Interestingly, only sympathetic denervation reduces the number and size of colon tumors induced by dimethylhydrazine, as well as the proliferation rate of cancerous cells [91] (Fig. 3).

However, in APC (min/+) mouse models, sympathetic denervation does not affect the number of tumors [92]. This finding suggested that the effect of these fibers is more significant in advanced stages of CRC than in early stages, such as ulcerative colitis. Consistent with this, β-adrenergic agonists increase the number of tumors induced with azoxymethane (**AOM**)/dextran sodium sulfate (**DSS**) in BALB/c mice. Additionally, in a xenograft mouse model, epinephrine increased epithelial–mesenchymal transition, pulmonary metastatic nodules, and tumor growth through the epinephrine-ADRB2 axis while reducing the expression of suppressor genes such as P53 [93] (Fig. 3).

Adrenergic receptors are expressed in both innate and adaptive immune cells, and their activation increases the concentration of kinases that modulate the expression of factors such as NFκB, Ras, and Src, promoting survival, recruitment, and segregation of proinflammatory and anti-inflammatory factors [94]. As mentioned above, denervation of the sympathetic nervous system prevents the progression of colon tumors. One possible explanation is the immunosuppressive effect of adrenergic pathway activation on immune cells, which could prevent the killing of tumor cells [95]. For example, it decreases phagocyte capacity and the secretion of TNF-α in macrophages; reduces the maturation, cytotoxicity, and migration of natural killer cells and neutrophils; and, in the adaptive response, prevents the cytotoxicity of CD8 + cells and antibody production, reduces IL-2 synthesis, and upregulates the suppressive actions of regulatory T cells [96] (Fig. 3).

**Effect of sex steroids on the nervous system**.

The sympathetic nervous system is associated with the response to dangerous or stressful events. This system modulates the function of many tissues, such as the eyes, heart, lungs, stomach, intestines, bladder, kidneys, and salivary glands, through adrenergic receptors [97]. Interestingly, E2 has an inhibitory effect on this system in many tissues. For example, postmenopausal women have greater sympathetic activity and blood pressure than young women, and E2 inhibits this effect [98]. Treatment of insulin-resistant rats with E2 has a cardioprotective effect; it reduces heart rate, prevents arterial pressure, and diminishes the vasopressor muscular response [99]. In mice, E2 suppresses renal ischemia, and this effect is inhibited by tamoxifen [100]. Reconstituted OVX rats exhibit decreased blood pressure, heart rate, and nervous renal activity [101]. Interestingly, men treated with estradiol and exposed to a stressful event showed an increase in acetylcholine (Ach) and cortisol and increased activity in the HPA axis, suggesting that E2 has a dimorphic effect on the modulation of the sympathetic nervous system [102]. This leads us to think that E2 exerts an inhibitory effect on the SNS in the colon, which causes a diminished protumoral effect.

In murine models, E2 enhances neurogenesis in damaged enteric neurons and increases the proliferation of enteric glial cells through interaction with the ER-β. Dysfunction of glial cells in the enteric system is a consequence of cancer invasion, and these damaged nerves release prosurvival and proliferation signals that favor a microenvironment promoting tumor growth; for example, Ach indirectly activates the MAPK and PI3K pathways, favoring proliferation [103]. These findings suggested that estrogens promote the inhibition of tumor formation by stimulating neurogenesis and thereby preventing the release of prosurvival factors. On the other hand, estrogens also regulate the production of serotonin (5-HT) at different levels. For instance, the number of neurons that release 5-HT is reduced in the left ventricle of ovariectomized macaques [104].

Similarly, the synthesis of 5-HT in the brain neurons of macaques is favored by E2, and these hormones also promote the expression of the serotonin reuptake transporter. Finally, E2 stimulates the expression of 5-HTR in the brains of postmenopausal women [105]. Interestingly, 5-HT exerts a protumoral effect on colorectal cancer progression. One explanation is that E2 exerts a different effect on enteric neurons than on brain neurons or that it favors the release of other antitumoral factors, such as Ach. In line with this idea, the administration of E2 enhances the production of Ach through 5-HT interactions in the frontal cortex of mice [106]. However, additional studies are needed to elucidate the role of these hormones in the enteric system and their relationship with the pathophysiology of colorectal cancer.

The stress response is modulated by the sympathetic nervous system, which regulates heart rate, blood pressure, and the release of epinephrine and adrenaline. Several studies have shown that androgens activate this system. For instance, men have higher blood pressure than women of the same age [107]. Castrated male rats exhibit lower levels of hypertension than control animals, and flutamide decreases blood pressure to the level found in females [108]. Additionally, women with polycystic ovary syndrome, which is characterized by elevated T levels, exhibit hypertension, and OVX mice treated with T experience an increase in blood pressure [108]. Interestingly, the administration of T reduces norepinephrine in plasma but increases it in myocardial tissue, and this effect is inhibited by reconstitution with T. Furthermore, castrated rats exhibit a 22% decrease in nerve density [109]. All of these findings suggest that androgens stimulate nerve innervation and the release of neurotransmitters, which could stimulate tumor progression.

Regarding the role of androgens, pubertal mice exhibit high expression of AR in enteric neurons. Experiments with ORX mice have shown that androgens participate in gut motility; however, they do not affect the density of enteric neurons. This finding suggested that androgens affect the physiology and mechanisms of action rather than the survival or genesis of neurons [110]. Similarly, castrated ORX macaques reconstituted with T and DHT exhibit a higher concentration of serotonin transporter in their dorsal raphe [111]. Although healthy males and females do not differ in the expression of ARs in enteric neurons, men have higher levels of androgens, which may explain why males synthesize 52% more serotonin than females across all brain areas [112]. This finding suggested that androgens stimulate enteric neurons to secrete serotonin, creating a protumoral microenvironment. However, androgens also regulate the expression of Ach. Orchidectomy impairs the release of Ach in the hippocampus of rats and affects the transcription of Ach receptor mRNA [113, 114]. However, additional studies are necessary to elucidate its relationship with diseases such as colorectal cancer.

The principal soluble factors of the enteric nervous system (5-HT and Ach) can modulate the immune response. In AOM/DSS models, 5-HT exacerbates the induction of colitis through an inflammatory response mediated by proinflammatory cytokines. For example, in the colon of DSS-induced colitis mice, 5-HT favors the release of IL-12 from DCs, which activates T cells that produce IL-17 and IFN-γ. Additionally, in the same mouse model, macrophages express high levels of the 5-HT receptor, and a specific antagonist of this receptor inhibits the synthesis of IL-12 and iNOS [89]. Other models have shown that 5-HT exacerbates the functions of innate and adaptive immune responses. For instance, it increases phagocytosis in macrophages, enhances cytolytic activity in NK cells, acts as a chemotactic agent for eosinophils, and increases the activation of T lymphocytes. Furthermore, lymphocytes are capable of producing 5-HT, and DCs can serve as reservoirs for this molecule, potentially causing a self-sustaining paracrine response [115]. Taken together, these findings suggest that serotonin participates in the early stages of CRC development and promotes chronic inflammation, allowing the accumulation of mutations in neoplastic cells and the disruption of colon tissue architecture (Table 2).

**Table 2:** Modulatory effects of sex steroids on nerve fibers and their metabolites. Arrow up indicates stimulation and arrow down inhibition

Sex steroids	Effect
**Estradiol** [98–100, 103, 104, 106, 108]	⇧neurogenesis in damaged enteric neurons, cell proliferation of enteric glia cells through their interaction with ER-β. ⇧enteric neurons damaged. ⇧sympathetic activity in postmenopausal women and blood pressure than young women. ⇩activation of sympathetic nervous system. ⇩ blood pressure, heart rate, nervous renal activity, ⇧ Ach, 5-TH in brain (no data in colon).
**Androgens** [107, 109–114]	⇧expression of AR in enteric neurons of pubertal mice, in ORX mice no affect the density of enteric neurons, ⇧sympathetic nervous system, ⇧levels of 5-HT in blood in men, ⇧ hypertension, norepinephrine in heart, nerve density. ⇧SERT and Ach

#### Microbiota and colorectal cancer

The intestinal microbiota is composed of a large population of microorganisms, primarily bacteria from the Firmicutes and Bacteroidetes phyla. These microorganisms play a crucial role in physiological processes such as protection against pathogens, regulation of the immune response, and provision of energy to colonocytes. Dysbiosis is defined as a reduction in microbial diversity, the loss of beneficial bacteria that secrete short-chain fatty acids, and an increase in facultative anaerobic bacteria such as Proteobacteria [116]. Imbalances or disturbances in the gut microbiota or associated patterns have been recognized as indicators of certain diseases or poor health status. Dysbiosis occurs in the early stages of colorectal cancer (CRC) and favors processes such as inflammation, DNA damage, and the avoidance of cancer cell death. However, it is still unclear whether this phenomenon promotes inflammation or is a consequence of itself [117, 118]. Specifically, patients with CRC have lower bacterial diversity than healthy individuals [119].

At the species level, some bacteria are associated with the development of this disease, and the presence of these diseases depends on the stage of cancer. For example, the concentrations of *Solobacterium moorei, Peptostreptococcus stomatis, Peptostreptococcus anaerobius, Lactobacillus sanfranciscensis, Parvimonas micra*, and *Gemella morbillorum* continuously increase throughout the tumor, and their abundance is associated with a poor prognosis. Other species, such as *Atopobium parvulum, Actinomyces odontolyticus, Desulfovibrio longreachensis*, and *Phascolarctobacterium succinatutens*, are present in the early stages, while *Collinsella aerofaciens, Porphyromonas uenonis*, and *Dorea longicatena* increase in number only in the advanced stages [120].

On the other hand, there is direct evidence of the role of certain species. Mice that spontaneously develop adenomas and are fed *Fusobacterium nucleatum* develop colitis more rapidly through the interaction of the FadA receptor in bacteria with E-cadherin, triggering the activation of the β-catenin pathway, which promotes the proliferation and transformation of cells toward a neoplastic state [121]. The abundance of *Enterococcus faecalis*, which is increased in neoplastic mucosa, induces the production of reactive oxygen species, causing DNA damage [122, 123]. Additionally, some beneficial bacteria that produce SCFAs, such as *Clostridium butyricum*, are absent in the mucosa of CRC patients [124].

The gut microbiota can affect tumor growth through the regulation of the inflammatory response. Fecal transfer from colorectal cancer (CRC) patients to germ-free mice increases the production of histological inflammation and inflammatory factors, including chemotactic factors that promote a protumoral TH1 response. *Fusobacterium nucleatum* increases the infiltration of proinflammatory myeloid cells, enhancing intestinal tumorigenesis. The effect on immune cells is mediated mainly by the release of short-chain fatty acids (SCFAs), primarily butyrate. For example, butyrate promotes an immunosuppressive microenvironment by enhancing the polarization of macrophages toward the M2 phenotype, reducing the production of proinflammatory cytokines by dendritic cells, and increasing the number of regulatory T cells in vivo [125, 126]. CR patients have lower levels of butyrate and its receptor [127]; this metabolite is capable of inducing proliferation in normal epithelial colon crypts while inducing apoptosis in colon adenoma and neoplastic cells [128, 129]. The above findings suggest that in the early stages, the loss of bacteria that produce butyrate promotes chronic inflammation and induces the proliferation of cancer cells, facilitating the initiation of carcinogenesis.

#### Effect of sex steroids on the modulation of the microbiota

Sex steroids have a close bidirectional relationship with microbiota physiology and dysbiosis. Androgens and estrogens undergo conjugation through glucuronidation and sulfation in the liver and are subsequently excreted into the biliary tree and the intestine. In the bowel, certain bacteria synthesize enzymes such as β-glucuronidase, hydrolases, and sulfatases, which deconjugate these hormones; additionally, *E. coli* is capable of producing androgens through bile acids, thereby increasing the reuptake and bioavailability of sex steroids [130].

Compared with premenopausal women, postmenopausal women experience a significant reduction in the diversity of the microbiota. Furthermore, these women exhibited an increase in Bacteroidetes and a decrease in Firmicutes. This alteration in the composition of the microbiota is associated with elevated levels of inflammatory factors such as MCP-1 and IL-6 ^131,132^. It seems that the effect of this treatment depends on ER-β; intestinal ER-β-deficient mice fed a high-fat diet exhibit decreased microbiota diversity, lower levels of Bacteroidetes, and greater levels of anaerobic bacteria, which are associated with a poor prognosis in colorectal cancer (CRC) patients [133]. Similarly, β-estrogen receptor knockout (BERKO) mice treated with AOM/DSS to induce intestinal tumorigenesis exhibited decreased microbiota diversity and an increase in gram-negative proinflammatory bacteria [134].

Furthermore, a study conducted on male and female ICR mice revealed that females, control males treated with E2, and AOM/DSS/E2-treated males had greater microbiota diversity than control mice. Interestingly, the Firmicutes/Bacteroidetes ratio decreased only in AOM/DSS-treated males supplemented with E2, and this group exhibited a lower tumor grade. This finding suggested that E2 protects males by reducing dysbiosis and preventing chronic inflammation [135]. These studies suggest that estrogens protect against colorectal cancer by reducing chronic inflammation through the reduction of proinflammatory bacteria and by maintaining a high diversity of these microorganisms. However, another possibility is that estrogens impact the immune system, triggering an anti-inflammatory response and preventing chronic inflammation and dysbiosis.

Androgens have a direct influence on the microbiota composition in both men and women. Studies of the metagenome have shown a clear difference in the microbiota composition between men and women. This difference is called the microgenderome. For example, females exhibit greater microbial diversity and a greater richness of antibiotic resistance genes [136]. Compared with females, males have a greater abundance of *Prevotella, Megamonas, and Fusobacterium* [137]. Multiple external factors, such as the environment, habits, and diet, could influence these differences; however, prepubertal mice show no difference in microbiome composition between males and females. The composition of the microbiota is influenced during puberty. In particular, females have greater alpha diversity, and this effect is reversed by the castration of males [138].

The microbiota and androgens have bidirectional interactions since some bacteria can regulate androgen biosynthesis. The gut microbiota degrades intestinal glucuronidated T and DHT from the liver, increasing the bioavailability of androgens. Interestingly, the fecal DHT concentration in the distal intestine was 70-fold greater than that in the serum of young men [139]. In a very broad sense, studies suggest that estrogens favor an antitumoral effect by protecting the diversity of bacteria and promoting the growth of species that maintain intestinal homeostasis, while androgens reduce this diversity and favor the growth of bacteria that promote the inflammatory process, thus contributing to carcinogenesis (Table 3).

**Table 3:** Bidirectional interaction between the intestinal microbiota and sex steroids

Sex steroid	Effect on Microbiota
**Estrogens** [130–135]	⇧reuptake and bioability of E2 by enzymes (produced by bacterias) in bowel that deconjugate E2 glucurodinated ⇧E2 ⇩microbial diversity (in postmenopausal women), inflammatory factors, effect through ER-β ⇩microbiota diversity⇧progression of colon tumors.
**Androgens** [136–139]	⇧reuptake and bioability of androgens by enzymes (produced by bacterias) in bowel that deconjugate T glucurodinated, ⇧Fecal DHT concentration in the distal intestine (70-fold) ≥ serum from young men, ⇧Androgens ⇩ diversity, effect is reversed by the castration of males.

#### The effects of sex steroids on the neuroimmune network during colorectal cancer development

**Fig. 4:**
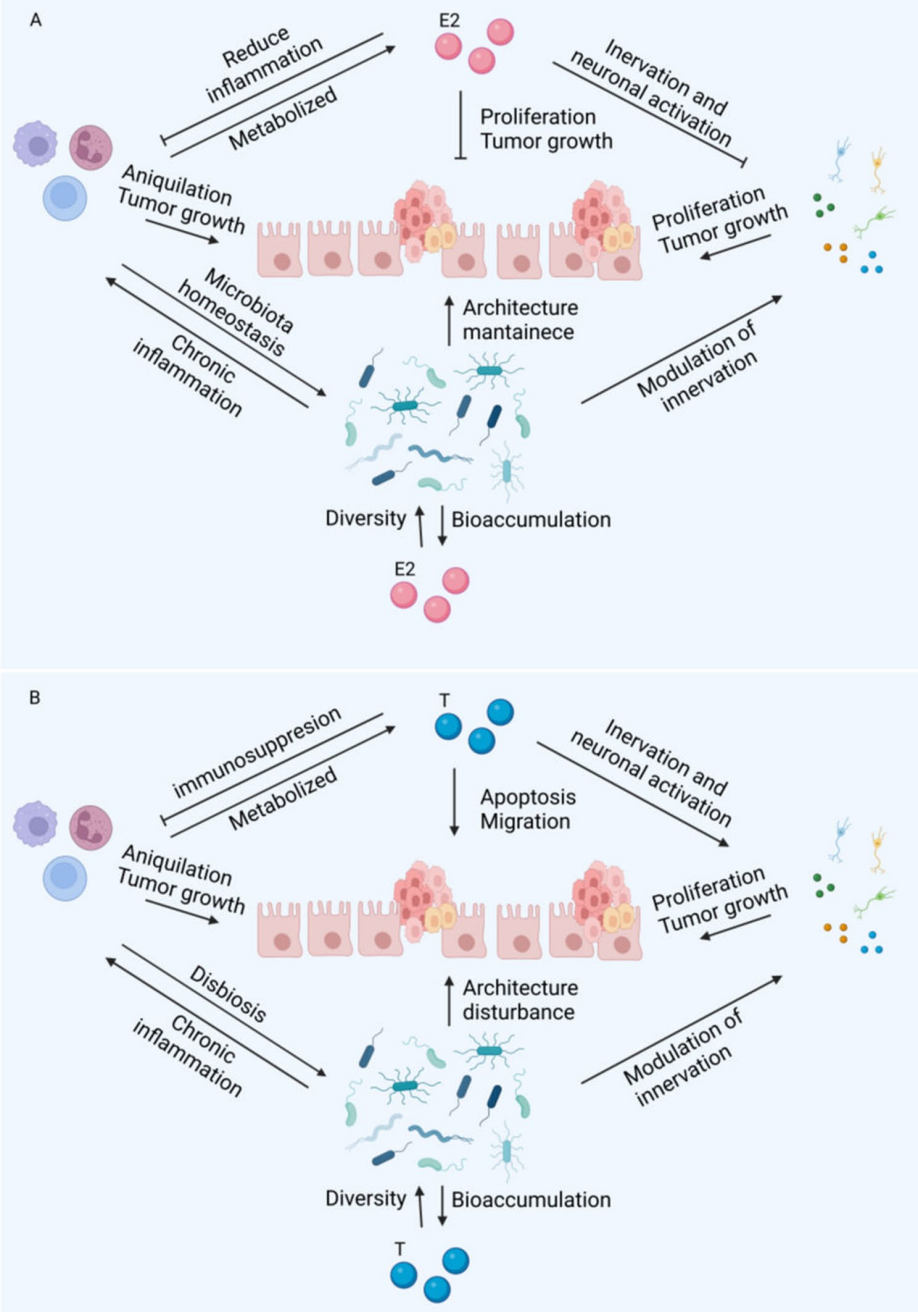
Regulation of sex steroids to neuroinmmunedocrine network. E2 potentially inhibits tumoral progression by several mechanisms. First, this hormone avoids chronic inflammation; affects the enteric system. Primarily, it stimulates neurogenesis and maintains tissue architecture. Also, increase the diversity of bacteria which protects against the development of tumors. Finally, E2 reduce the proliferation of tumor cells directly through their interaction with ER-β (**A**). Possible regulatory effects of androgens on the neuroendocrine network and repercussions for tumor growth. Contrarily, androgens play an immunosuppressive role that potentially help tumor cells to evade immune response. These molecules stimulate the release of catecholamines, 5-HT and Ach that promotes tumor growth and invasion. Additionally, androgens reduce the diversity of bacteria in the microbiota. Finally, stimulate apoptosis and migration of tumor cells directly (**B**). T: testosterone. E2: estradiol. This figure was created with BioRender.com

Since CRC is strongly associated with chronic inflammation, we analyzed the effect of sex steroids on cells in the colon microenvironment (immune cells, neurons, and bacteria). In general, E2 have an antitumoral role (Fig. 4A) and androgens a protumoral (Fig. 4B). The possibly mechanisms based on the studies analyzed in this review are represented in Fig. 4.

Interestingly, depending on the dose, E2 has an anti-inflammatory effect on the innate immune response, especially in studies that directly assess inflammation in colonic epithelium tumors. However, it is important to mention that estrogens can activate dendritic cells and lymphocytes, which are essential cells for tumor elimination, and their infiltration into the microenvironment is clearly associated with a favorable prognosis. These findings suggest that this hormone potentially protects the intestine against damage by preventing chronic inflammation in the early stages of carcinogenesis. Nevertheless, it would be interesting to conduct studies that evaluate the effect of this hormone in advanced stages (Fig. 4A).

E2 affects the enteric system. Primarily, it stimulates neurogenesis and maintains tissue architecture. Damage to enteric glial cells is a consequence of cancer invasion, and this damage leads to the release of prosurvival and proliferative signals that create a microenvironment favorable for tumor growth. For example, Ach indirectly activates the MAPK and PI3K pathways, promoting proliferation. Furthermore, although the effect of the microbiota is complex, E2 increases bacterial diversity, which is associated with a good prognosis in patients with colorectal cancer (CRC) and reduces tumor progression in animal models. This effect is dependent on ER-β. On the other hand, E2 inhibits the activation of the sympathetic nervous system and the secretion of factors that promote tumor growth, thereby favoring an antitumoral role (Fig. 4A).

Animal studies have shown that the protective effect of E2 on the development of colonic tumors is weaker in females and stronger in males. The above findings open the possibility of estrogen analog treatment in men. Although aromatase and E2 are overexpressed in the colon of men with colorectal cancer compared to healthy individuals, there is also a decrease in ER-β expression and an increase in ER-alpha expression. Therefore, alternative approaches for enhancing the transcription and expression of ER-β should be explored. Like estrogens, androgens potentially modulate immune, nervous and microbiota cells in the tumor microenvironment of the colon (Fig. 4A).

In general, androgens play an immunosuppressive role in both the innate and adaptive immune systems. These findings suggested that these molecules help tumors evade immune responses. Additionally, although studies suggest that androgens do not alter neurogenesis, they are capable of stimulating the synthesis of molecules such as those in the sympathetic nervous system (adrenaline and noradrenaline), 5-HT, Ach, and their receptors. These molecules promote tumor cell proliferation, tissue degradation, tumor formation, and the production of angiogenic factors such as MM-9 and 3. Furthermore, they stimulate the secretion of IL-12, IL-17, and IFN-γ, which potentially promote carcinogenesis through chronic inflammation. Interestingly, the expression of AR in enteric neurons did not differ between healthy males and females. However, men have higher levels of androgen, which could explain why males synthesize 52% more serotonin than females on average across all brain areas. This finding suggested that androgens stimulate enteric neurons to secrete serotonin, favoring a protumoral microenvironment. It is also possible that increased sympathetic innervation and the release of norepinephrine promote tumor growth. In addition, males have a lower diversity of microbiota than females, and animal models suggest that androgens favor a decrease in biodiversity, while the microbiota increases the bioavailability of androgens in the colon. Decreased diversity is a risk factor associated with poor prognosis in the early stages of CRC carcinogenesis [69] (Fig. 4B).

The idea that androgens promote a protumoral response is supported by studies with animals in which gonadectomized animals develop fewer and smaller tumors, and the effect is reversed by the administration of DHT and, to a lesser extent, by T. Additionally, in vitro studies of Dutasteride and other inhibitors of Steroid 5α-reductase type I, which in turn converts testosterone to dihydrotestosterone, revealed accelerated cell apoptosis and reduced cell proliferation and migration in the colorectal cancer cell lines HCT116 and LOVO [140]. Additionally, CRC patients have higher expression of androgen receptors in tumors than in healthy tissue. Notably, E2 does not protect against tumor development, but androgen removal has such a potent effect. However, it is important to highlight that the effect of androgens has been tested only in male organisms. It would be very interesting to evaluate their role in females, as these molecules are also of great importance in the physiology of the colon in females.

#### Hormonal therapy

The evidence of the effect of sex steroids on the development of colorectal cancer (CRC) is limited to studies with postmenopausal women. In vivo, studies have not shown as potent a tumor-promoting effect of endogenous estrogens as androgens. Exogenous hormones in the form of hormone replacement therapy (estrogens plus progestins) had a protective effect on almost 45% of the women in this group compared to those in the placebo group. However, one drawback of this type of treatment is the increased incidence of breast and endometrial cancer in women [141]. On the other hand, the possibility of using phytoestrogens has increased. These compounds can mimic the effect of estrogens and have beneficial effects on symptoms in postmenopausal women as well as on breast cancer [142]. Epidemiological data show that the consumption of foods containing a high concentration of these molecules reduces the incidence of CRC in both men and women, suggesting that consuming these foods is a good alternative for possible treatment [143].

The use of sex steroids, agonists, or antagonists of these molecules as treatments for CRC in humans is currently unexplored. Interestingly, in vitro and in vivo studies have shown that these molecules could have great potential for a new age of treatment for CRC. Based on the information analyzed, the most potent hormone involved in the development of CRC in males is DHT. The use of antagonists of this hormone is a potentially effective treatment. For example, the use of older drugs such as flutamide and newer generation antiandrogens such as enzalutamide and apalutamide, which prevent the translocation of the AR from the cytoplasm to the nucleus through competitive inhibition, now plays a major role in the treatment of metastatic castration-resistant prostate cancer [144]. To date, the effect of androgens has been tested only in males, and it is necessary to conduct studies in both sexes to assess whether they have the same tumor-promoting effect on male and female patients.

Another possibility is the use of ER-β-specific agonists or phytoestrogens since studies have demonstrated that ER-β has an antitumor effect both in vivo and in vitro and is associated with a good prognosis in humans (as mentioned in the previous section). For example, an interesting proposal would be the use of diarylpropinitrile, which inhibits the growth of MC38 colon cancer cell lines in vitro [145]. Moreover, in breast cancer, ER-β plays a protective role against the growth of primary tumors and metastasis [146]. In AOM/DSS-treated germ-free C57BL/6 mice, evodiamine (a phytoestrogen) reduces the number and size of colon tumors. Treatment also reduces proliferation, increases apoptosis, and restores the microbiota to a basal state in colon tissue [147].

Due to the limited number of studies, the use of sex steroids and their analogs against CRC is a field with much potential that must be explored more extensively in vivo to apply translational medicine and develop medical treatments for humans. All these potential strategies have the advantage that similar molecules are naturally produced by the human body and should be used in combination, considering sex.

## Conclusions

In general, estrogens play an antitumoral role in the development of colorectal cancer. Primarily, estradiol reduces proinflammatory immune molecules, leading to a decrease in chronic inflammation and an increase in microbiota diversity, which is associated with an antitumoral response. Moreover, these molecules inhibit the activation of the sympathetic nervous system, potentially contributing to their antitumoral effect.

On the other hand, androgens have a protumoral role. These hormones increase the release of anti-inflammatory molecules and promote a protumoral phenotype in immune cells. This potentially inhibits the cytotoxic response against tumors and reduces the biodiversity of the intestinal microbiota. Additionally, androgens activate the sympathetic and enteric nervous systems, as well as the release of serotonin, acetylcholine, and catecholamines, all of which have a protumoral role in colon tumor growth.

Notably, despite all the evidence of the role of steroid sex hormones in colorectal cancer progression, the conclusions of these studies indicate the carcinogenic properties of testosterone and dihydrotestosterone (DHT) and the protective effect of estradiol. To design an effective therapy and improve patient outcomes, it is necessary to account for all the factors that impact the homeostasis of the colon, starting with the sex of the patient, the age of the patient, the levels of the steroid hormones circulating, and the comorbidities to first restore healthy conditions in the colon and consider all the risk factors to provide a more holistic approach.

## Data Availability

Not applicable.

## Abbreviations

Ach: Acetylcholine
AOM: Azoxymethane
AR: Androgen receptor
CRC: Colorectal cancer
DHT: Dihidrotestosterone
DSS: Dextran sulphate of sodium
E2: Estradiol
5-HT: Serotonin
ER: Estrogen receptor
ORX: Orchiectomy
OVX: Ovariectomy
ROS: Reactive species of oxygen
T: Testosterone
